# Network Analysis Performed on Transcriptomes of Parkinson’s Disease Patients Reveals Dysfunction in Protein Translation

**DOI:** 10.3390/ijms25021299

**Published:** 2024-01-21

**Authors:** Simone D’Angiolini, Maria Lui, Emanuela Mazzon, Marco Calabrò

**Affiliations:** IRCCS Centro Neurolesi “Bonino-Pulejo”, Via Provinciale Palermo, Contrada Casazza, 98124 Messina, Italy

**Keywords:** Parkinson’s disease, protein synthesis, transcriptomic data, peripheral blood, network analysis

## Abstract

Parkinson’s disease (PD) is a prevalent neurodegenerative disorder characterized by the progressive degeneration of dopaminergic neurons in the substantia nigra region of the brain. The hallmark pathological feature of PD is the accumulation of misfolded proteins, leading to the formation of intracellular aggregates known as Lewy bodies. Recent data evidenced how disruptions in protein synthesis, folding, and degradation are events commonly observed in PD and may provide information on the molecular background behind its etiopathogenesis. In the present study, we used a publicly available transcriptomic microarray dataset of peripheral blood of PD patients and healthy controls (GSE6613) to investigate the potential dysregulation of elements involved in proteostasis-related processes at the transcriptomic level. Our bioinformatics analysis revealed 375 differentially expressed genes (DEGs), of which 281 were down-regulated and 94 were up-regulated. Network analysis performed on the observed DEGs highlighted a cluster of 36 elements mainly involved in the protein synthesis processes. Different enriched ontologies were related to translation initiation and regulation, ribosome structure, and ribosome components nuclear export. Overall, this data consistently points to a generalized impairment of the translational machinery and proteostasis. Dysregulation of these mechanics has been associated with PD pathogenesis. Understanding the precise regulation of such processes may shed light on the molecular mechanisms of PD and provide potential data for early diagnosis.

## 1. Introduction

Parkinson’s disease (PD) ranks as the second most prevalent neurodegenerative disorder after Alzheimer’s disease, reaching ~8.5 × 10^3^ cases worldwide [1]. The clinical phenotype of this pathology is characterized by a distinctive pattern of impairment involving primary motor symptoms, secondary motor symptoms, and non-motor symptoms [2]. The motor syndrome is characterized by movement dysfunctions like slow movement (bradykinesia), reduced movement (hypokinesia), or even an inability to move (akinesia). Another distinctive motor feature of PD is the resting tremor, like trembling or shaking of the hands, but also affecting limbs, legs, feet, the face, and arms [3]. Patients suffering from this multifactorial disease also experience secondary motor symptoms such as gait rigidity, postural instability, dystonic cramps, dysphagia, dysarthria, together with impaired balance and coordination, which drive the progression of the motor disability over time [4]. This progressive neurodegenerative disorder also occurs with a broad spectrum of non-motor symptoms including autonomic dysfunction (hyposmia, constipation, urinary dysfunction, orthostatic hypotension), cognitive/neurobehavioral disorders (memory loss, depression, dementia pain), and sensory and sleep abnormalities [5]. The causative factors at the basis of PD are still under study since it is a pathologically and genetically heterogeneous disease with a multifactorial etiology based on environmental and genetic factors. More than 100 genes have been identified to be involved in PD etiology [6], but most of them are likely to explain only a small portion of PD and about 90% of cases are related to the sporadic form [7]. Considering the previously reported studies, there is an active field of research around PD to better clarify the molecular mechanisms underneath the disease and eventually improve current therapeutic strategies [8]. In this context, the use of PD samples deposited on publicly available datasets could be a useful resource to expand the current knowledge about this neurodegenerative disorder [9]. Nowadays, the large amount of deposited whole blood and peripheral blood mononuclear cell expression data of PD patients have raised great interest in the analysis of the transcription profiles of peripheral tissues. Several studies on expression profiles of PD patients revealed common pathways altered in both brain and blood samples, suggesting the latter as a valid tissue to evaluate the state of the disorder [10]. Indeed, this moved the interest for the investigation of the transcriptome of PD at the peripheral blood level [11], which can represent a valid and non-invasive approach for not only clarifying the disease-leading mechanism, but also for diagnosis and prognosis purposes and monitoring therapeutic responses [12]. Multiple pathways and biological processes have been found to be altered in PD and have been proposed to play a key role underneath the disease. This includes proteostasis [13], whose alteration is often linked to various neurodegenerative diseases. In recent years, numerous studies have been conducted in this direction to elucidate the actual interplay between proteostasis and PD [14,15]. To gain deeper insights into this process, the current study aimed to investigate the dysregulation of elements involved in the proteostasis-related mechanism at the transcriptomic level. This exploration was conducted using a sample of blood tissues from PD patients sourced from a publicly available dataset deposited in the NCBI Gene Expression Omnibus (GEO) database [16].

## 2. Results

**Figure 1:**
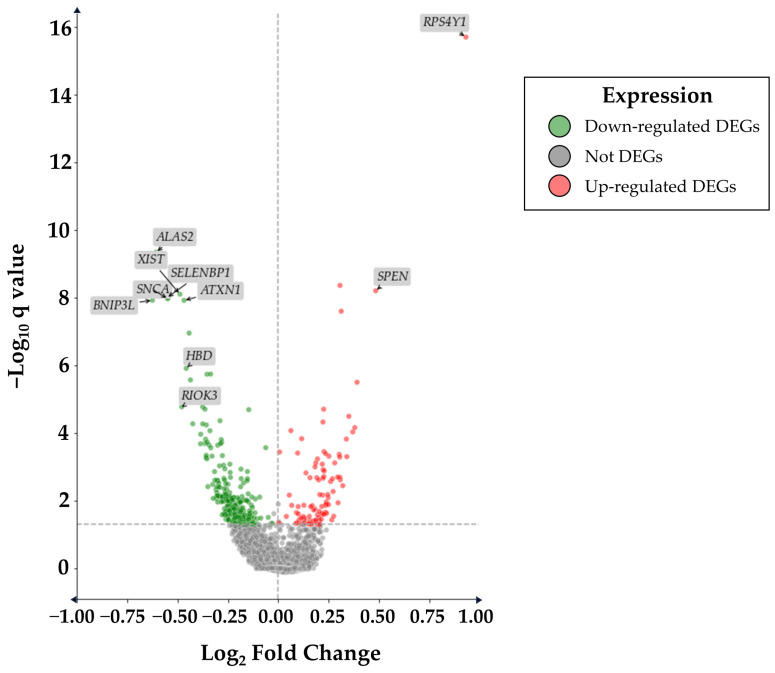
In the dot plot are reported all the features explored in the analysis. The line that intercepts *y* axis is related to our threshold of significance of q value (0.05), all the genes above this line are considered differentially expressed. In the *x* axis is reported the log_2_ fold change that discriminate up and down-regulated DEGs defined by red or green color respectively.

In the present study, we analyzed gene expression data from blood samples of healthy controls (HC) and PD patients using the rank product method to identify differentially expressed genes (DEGs). Starting from the complete dataset that included 15,786 probes, our analysis revealed a total of 375 DEGs, among which 281 identified as down-regulated and 94 as up-regulated. The terms up-regulated refer to the DEGs that have a statistically confirmed increase in the PD sample, wherein the down-regulated DEGs are less expressed in PD compared to HC. The statistical validity of our DEGs was confirmed by adjusting *p* values using the false discovery rate (FDR) method; this approach allowed us to carefully control the false positive rate. DEGs with an FDR-adjusted *p* value (q value) < 0.05 were considered statistically significant. In order to attain the most comprehensive perspective possible and to understand potential alterations across entire gene ontologies, no filters based on fold change were applied. The results about the number of up-regulated, down-regulated, and non-significative altered genes are shown in Figure 1.

**Figure 2:**
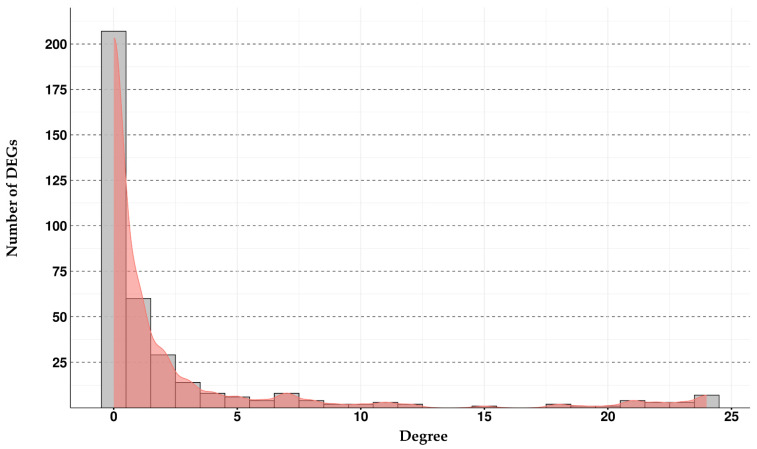
In the histogram are reported, on the *x* axis, the degree scores from the lowest (0) to the highest (24), while in the *y* axis is reported the number of DEGs for each degree value.

Scores related to the fold change, *p* value, and q value of each gene are available in Supplementary Table S1. A comprehensive analysis was undertaken by uploading the entire set of DEGs onto the STRING database to elucidate potential interactions among our identified DEGs. Advanced settings were configured using the highest confidence level, 0.900, and the maximum allowable FDR, 1%. These parameters were selected to ensure a robust depiction of the network, emphasizing connections between nodes that could be deemed highly reliable. By opting for the highest confidence threshold and the strictest FDR stringency, we aimed to attain a secure and trustworthy representation of the network topology, thereby enhancing the credibility of identified interactions. This approach was undertaken to provide a comprehensive and reliable overview of potential relationships within the biological system under investigation. The result is a network composed of 371 nodes and 453 edges. Analysis of the network allowed us to obtain, for each node, information about different parameters. All the information about the nodes of our network can be consulted in the Supplementary Table S2. We used the score of degree to filter the DEGs of our network and isolate those ones with the highest levels. Score of degree indicates the number of edges that connect a node to other nodes; higher degrees correspond to more connected nodes among the network. In Figure 2 is shown the histogram with the distribution of the number of DEGs for each score of degree.

**Figure 3:**
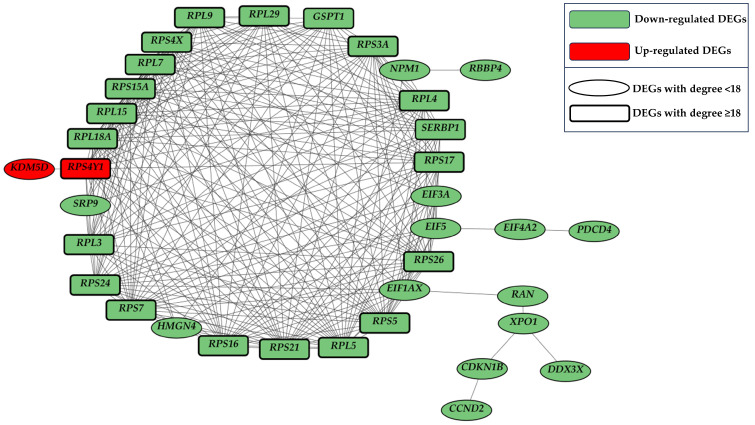
Subnetwork composed of 36 DEGs connected through 266 edges. In green are reported the down-regulated DEGs in PD, while in red are reported the up-regulated ones. The DEGs with a rectangular shape and thickened edges are the 21 with a value of degree ≥ 18.

As depicted in Figure 2, it is evident that the majority of DEGs exhibit a degree score near 0. Specifically, the number of isolated DEGs, denoting those with no connections and possessing a degree score of 0, amounts to 207. Notably, an intriguing observation pertains to the group of DEGs related to the higher degree scores. In this context, 21 DEGs fall within a degree value between 18 and 24. Considering the relevant score of degree, these 21 DEGs underwent further scrutiny to assess their potential involvement in specific subnetworks. The 21 DEGs with a degree ≥ 18 are “*GSPT1*”, “*RPL15*”, “*RPL18A*”, “*RPL29*” “*RPL3*”, “*RPL4*”, “*RPL5*”, “*RPL7*”, “*RPL9*”, “*RPS15A*”, “*RPS16*”, “*RPS17*”, “*RPS21*”, “*RPS24*”, “*RPS26*”, “*RPS3A*”, “*RPS4X*”, “*RPS4Y1*”, “*RPS5*”, “*RPS7*”, “*SERBP1*”. Filtering these DEGs in the network, we discover that all of them are connected to each other through 207 edges with an average node degree of 19.7 and an average local clustering coefficient of 0.987. We diffused the subnetwork that included the 21 DEGs to better observe their context and we obtained a network composed of a total of 36 DEGs connected by 266 edges. This last network is shown in Figure 3.

**Figure 4:**
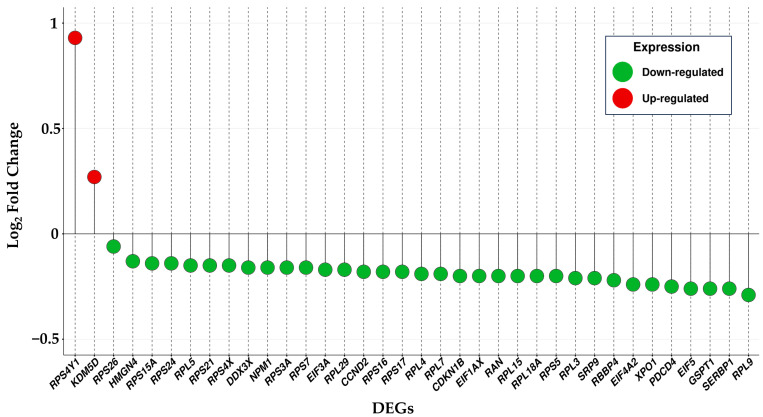
In the lollipop plot is reported the list of 36 DEGs shown in Figure 3 on the *x* axis and the associated log_2_ fold change on the *y* axis. Green dots are associated to down-regulated DEGs, and red dots to up-regulated DEGs.

Among the 36 DEGs reported in Figure 3, two were up-regulated and 34 down-regulated. The observed disparity in the number of up and down-regulated genes may indicate a generalized down-regulation linked to processes in which these DEGs could be involved. Prior to exploring the ontologies most pertinent to these DEGs, we assessed their log_2_ fold change levels, as depicted in Figure 4.

In order to ascertain the predominant gene ontologies (GO) within all the 375 DEGs in our network, we conducted an enrichment analysis for biological process (BP), cellular component (CC), and molecular function (MF). The genes associated with the enriched ontologies were cross-referenced with the 36 DEGs identified in the subnetwork illustrated in Figure 3. In pursuit of a comprehensive investigation, our analysis aimed to scrutinize transcriptional imbalances not only at the level of individual genes but also within the broader context of ontological categories. The enrichment analysis performed using all the 375 DEGs resulted in 276 BP, 63 CC, and 45 MF. We included as enriched all the GO with a q value < 0.05. All the enriched GO with associated DEGs can be retrieved in Supplementary Table S3. In Table 1 we reported all the enriched GO containing at least half of the DEGs from our focal subnetwork showcased in Figure 3.

## 3. Discussion

The multifactorial nature of PD makes it difficult to pinpoint the genetic background in action in the development of the disease and/or evaluate PD progression. In the present study, we tried to infer the dynamic changes in gene expression using a subset of subjects from the GSE6613 dataset [17,18]. Our aim was to clarify the processes likely altered in this disease and, possibly, suggest potential processes to be targeted to ameliorate PD symptoms or to be used as markers of PD progression. According to our results, 375 genes had a significant different expression in the blood tissues of PD patients compared to controls. The network analyses further identified a cluster of highly connected genes, as shown in Figure 3, which likely concur on the same/similar function. Interestingly, our results consistently point to a generalized impairment of the translational machinery and proteostasis. 

As a whole, proteostasis is fundamental for cell viability, maintenance, and response to stressors [19,20,21,22,23,24]. The deregulation of such process can be considered a physiological event observed with aging; a growing body of evidence also highlights that this decline is dramatically accelerated in neurodegenerative diseases, including in models of PD [13,21,25,26,27]. With “proteostasis” we identify the processes related to protein synthesis for accurate protein biogenesis, trafficking, folding, and removal [13]. A balanced proteostasis is essential for neuronal activity as 33–35% of newly synthesized proteins are prone to misfolding and aberrant protein accumulation was proven lethal for neurons [28,29]. Its dysregulation in the central neurons is apparent in PD pathology along with altered signaling events and mechanisms related to translational machinery [28,30,31]. Multiple factors are likely interconnected to preserve this balance, including regulation of protein synthesis, chaperone-assisted protein folding, and protein degradation pathways [32,33]. 

Our analysis highlighted a group of dysregulated genes mainly involved in the protein synthesis processes, including translation initiation and regulation, ribosome structure, and ribosome components nuclear export (please refer to Supplementary Table S4). In detail, we observed a generalized down-regulation of genes whose products directly participate in the phases of translation (initiation phase, termination phase, and regulation of protein production). In particular, the eukaryotic initiation factors constitute the core element for translation initiation (*EIF1AX*, *EIF3A*, *EIF4A2*, *EIF5*). The function of these DEGs is finely regulated on multiple levels (isoforms interactions and cleaving), giving the related complexes a key role not only in translation but also in its regulation [34,35]. While no direct associations with PD were described in the literature, the down-regulation of these elements has been linked with the suppression of protein synthesis [36]. We observed the same alteration in our PD sample. The termination and protein trafficking also seem to be influenced. Our results evidenced a down-regulation of *SRP*, which encodes for a protein involved in the first steps of neo-synthesized proteins transport and correct localization to the endothelial reticulum [37,38,39]. A reduction in the levels of this element may lead to protein accumulation. Hernandez et al. observed that this element is involved in the regulation of αSyn biogenesis [40].

*SERBP1*, which encodes for a protein whose main role is to bind ribosomes to stabilize their structure and protect them from degradation, was found to be down-regulated in our sample. The ribosome is a cellular structure responsible for protein synthesis, and any disruptions in its components or function can impact the synthesis of proteins crucial for cellular health [21,41]. The down-regulation of *SERBP1* likely promotes the degradation of ribosomes, which may ultimately lead to a reduction in protein synthesis [42]. 

The consequences of *SRBP1* down-regulation are further potentiated by the reduction in the genes encoding for ribosome components’ expression (*RPL15*, *RPL18A*, *RPL29*, *RPL3*, *RPL4*, *RPL5*, *RPL7*, *RPL9*, *RPS15A*, *RPS16*, *RPS17*, *RPS21*, *RPS24*, *RPS26*, *RPS3A*, *RPS4X*, *RPS5*, *RPS7*). It should be noted that many of the components we found down-regulated have functions beyond protein biosynthesis that have been suggested in the literature; in particular, elements like *RPS15A*, *RPL3*, and *RPL7* are involved in the regulation of cell proliferation and cell death [43,44,45,46]. Their altered regulation may contribute to the dopaminergic cell degeneration commonly observed during PD pathogenesis [21,41].

A similar correlation with cell death was found for *NPM1*, *RAN*, and *XPO1*, which are mainly involved with nucleocytoplasmic transport; for *HMGN4*, *KDM5D*, and *RBBP4*, which participate in chromatin-modifying complexes; and for *CCND2*, *CDKN1B*, and *DDX3X*, which are mainly related to cell cycle control.

Regarding nucleocytoplasmic transport, this process comprises the multistep assembly, transport, and disassembly of protein and RNA cargoes entering and exiting nuclear pores. The literature data have implicated dysfunction in nucleocytoplasmic transport in various neurodegenerative diseases, including PD [47,48]. This dysfunction causes the dysregulation in nucleus-cytoplasm protein partitioning and influences proteostasis balance, ultimately promoting intracellular deposits of proteins and cargoes, another hallmark typical of neurodegeneration [49,50,51]. This accumulation is known to be toxic for cells and may represent a trigger for cell death and neurodegeneration [52]. Additionally, nuclear transport elements (NTE), in particular *NPM1*, which encodes for an NTE that acts as a chaperone, has the function of suppressing the aggregation of misfolded proteins [53]. The down-regulation we found in our sample may promote an increase of pathological proteins aggregation. 

Regarding the chromatin-modifying related DEGs evidenced by our analysis, they play a crucial role in the regulation of gene expression, slightly changing the overall accessibility of DNA from the replicative machinery, which ultimately leads to altered levels of proteins synthesis. Although *HMGN4*, *KDM5D*, and *RBBP4* have not been directly correlated with PD, the dysregulation of chromatin-modifying complexes has been implicated in various neurodegenerative diseases, including PD [13,54,55,56]. It should be noted that, according to the literature data, they seemingly act in regulating the balance between pro-apoptotic and cell survival signals. Data on these elements mainly refer to their role in carcinogenesis [57,58,59]. Nevertheless, some hypotheses can be drafted from this data: the down-regulation of *HMGN4* and *RBBP4*, as well as the up-regulation of *KDM5D* tend toward pro-apoptotic signaling. Protein encoded by *KDM5D* seems to act on E2F1 through demethylation, thereby suppressing cell growth and metastasis in some form of colorectal cancer [57]. Also, its suppression hampers cell apoptosis [60]. The overexpression we found in our data may suggest its potential role in triggering apoptotic signals in neuronal cells. Kugler et al. observed that increased expression of *HMGN4* leads to a decrease in the levels of various tumor suppressors [58]. Consequently, reducing its expression may trigger an opposite effect, promoting cell cycle arrest and apoptotic signaling. Finally, it has been observed that RBBP4 loss disrupts neural progenitor cell cycle regulation and leads to Tp53 acetylation and apoptosis [59].

The DEGs related to cell cycle control likely exert their influence on multiple processes, including cell survival: *CCND2* encodes for a cyclin2, whose main function, in complex with *CDK4* or *CDK6*, is to regulate and control the G1/S transition during the cell cycle. *CCND2* down-regulation has been associated with the suppression of cell proliferation [61] and pro-apoptotic mechanics [62,63]. The protein encoded by *CDKN1B* prevents the activation of cyclin E-CDK2 or cyclin D-CDK4 complexes, and thus controls the cell cycle progression at G1. *DDX3X* encodes for a member of the large DEAD-box protein family and its roles encompass transcriptional regulation, mRNA assembly, splicing, and export. Chen et al. evidenced how depletion of this element impairs neurite development [64] and, in addition, its down-regulation promotes apoptosis [65,66]. To date, the mechanism of action of these proteins in protein synthesis is not known. Further and more specific investigations are needed as no direct associations are known between them and the inhibition of protein synthesis. However, it should be noted that the promotion of cell cycle arrest and apoptosis usually correlates with a reduction in protein synthesis [67].

**Figure 5:**
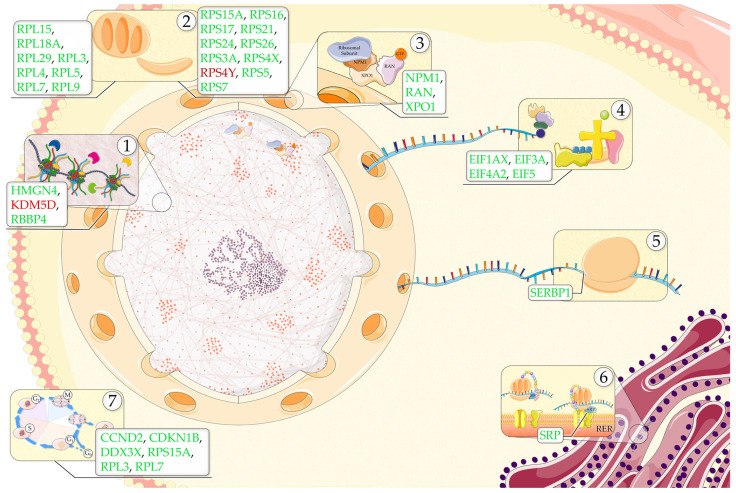
We summarized the processes linked to the DEGs we found in our analyses. In green we reported the down-regulated DEGs in PD patients, while in red we reported the up-regulated ones. (1) Chromatin Modifications (HMGN4 ↓, KDM5D ↑, RBBP4 ↓); (2) Ribosome Structure of 60S (RPL15 ↓, RPL18A ↓, RPL29 ↓, RPL3 ↓, RPL4 ↓, RPL5 ↓, RPL7 ↓, RPL9 ↓) and 40S (RPS15A ↓, RPS16 ↓, RPS17 ↓, RPS21 ↓, RPS24 ↓,RPS26 ↓, RPS3A ↓, RPS4X ↓, RPS4Y ↑, RPS5 ↓, RPS7 ↓) subunits; (3) Nuclear transport (NPM1 ↓, RAN ↓, XPO1 ↓); (4) 43S pre-initiation complex assembly and mRNA Cap Recognition (EIF1AX ↓, EIF3A ↓, EIF4A2 ↓, EIF5 ↓); (5) Ribosome Stabilization (SERBP1 ↓); (6) Signal Sequence recognition and Protein localization (SRP ↓); (7) Cell Cycle Control (CCND2 ↓, CDKN1B ↓, DDX3X ↓, RPS15A ↓, RPL3 ↓, RPL7 ↓). Parts of the figure were drawn by using pictures from Servier Medical Art. Servier Medical Art by Servier is licensed under a Creative Commons Attribution 3.0 Unported License (https://creativecommons.org/licenses/by/3.0/, 19 January 2024).

The global reduction in protein synthesis is not merely a consequence but potentially a conservative response of cells to stressors [24]. The complexity of this regulation involves a wide range of elements, with the literature data suggesting a significant contribution of PD-related genes to the overall control of protein synthesis. It has been observed that various genes related to PD, both dominant and recessive, such as *EIF4G*, *SNCA*, *LRRK2*, *PRKN*, *PINK1*, and PARK7, are connected to protein translation. The most apparent link is with *EIF4G1*. The protein encoded by *EIF4G1* works as a translation initiation factor and acts as a scaffold in the eIF4F translation initiation complex, recruiting ribosomes and tRNAs to mRNA, thereby playing a crucial role in protein synthesis [68]. Its alterations have been linked to PD [69] and play a role in the deregulation of protein translation [70]. *EIF4G1* is known to interact with *EIF1*, which was found down-regulated in our analysis, suggesting a possible common pathway of action. Regarding *SNCA*, it encodes for α-synuclein, a protein usually observed to form aggregates in the brains of PD patients. It has been shown that this protein may induce transcriptional repression through its interaction with ribosomal RNA and ribosomal proteins, leading to disruptions of ribosome function [71,72]. These interactions may contribute to the impairment of protein synthesis and the accumulation of misfolded proteins. Another notable player is the leucine-rich repeat kinase 2 gene (*LRRK2*), which is associated with both autosomal-dominant PD and the familial form of the disease [73,74]. The protein encoded by *LRRK2* has recently been show to play an active role in the global decrease in protein synthesis [75]. LRRK2 may intervene in regulating protein expression via multiple mechanisms, it has been observed that this protein interacts with several translation proteins, including many translation initiation factors [76] and ribosomal proteins [77], actively regulating protein synthesis. In addition, LRRK2 appears to regulate miRNA machinery [78], through which it further regulates protein expression levels by the inhibition of translation or the degradation of mRNA [79,80]. Another element likely associated with impaired protein synthesis is the protein encoded by *PINK1*. This protein acts in concert with the protein encoded by *PRKN* to regulate mitophagy [81,82]. It has been observed that *PINK1* is involved in the translation switch from cap-dependent to cap-independent translation during hypoxia, showcasing its role in adapting translation processes to cellular stress [83,84]. Finally, PARK7 (or DJ-1) is a redox-sensitive chaperone protein. When mutated, this protein has been shown to alter its affinity with ribosomal proteins, leading to decreased translation capacity [85]. In summary, our results support the presence of a global reduction in protein synthesis in PD and proteostasis imbalances, which likely translates in synaptic proteins loss, which in turn leads to eventual synaptic failure [41]. Also, the impairment of protein synthesis has been linked with the function of many PD genes, as described before. Many of these elements appear to play a regulative role in cell survival, as they are able to influence apoptotic pathways, and may take part in the pathogenesis of PD. In Figure 5 are reported all the DEGs presented with relative functions.

## 4. Materials and Methods

### 4.1. Microarray Dataset Selection 

A systematic search has been performed on publicly available databases GEO and ArrayExpress [86] to select a suitable dataset. Keywords used for the search were “Parkinson” and “Blood tissue”. All datasets were then filtered to retain only the ones containing transcriptomic data from microarray or RNAseq sources. To be selected, the datasets should include PD samples and control samples. Additionally, the dataset should comprise a sufficiently large number of samples to enhance the statistical significance of our analysis.

### 4.2. Dataset Description

Based on the previous section filter, we selected the data samples from the publicly available dataset deposited by Scherzer et al. [17,18] on GEO database with accession number GSE6613. In detail, we selected a subset of 72 samples, 50 PD patients, and 22 age-matched HC. According to the original authors, PD patients were eligible for the inclusion in the dataset if they met the criteria for PD according to the modified United Kingdom Parkinson’s Disease Society Brain Bank [87] clinical diagnostic criteria. To note, the United Kingdom Parkinson’s Disease Society Brain Bank criteria were changed to require at least three cardinal features, three supportive features, and none of the 16 exclusion criteria. Patients showed a mean age of 69.4 ± 8.4 years, with a 78% male ratio. Also, patients included in the dataset did not take any therapeutic treatment for PD. Clinical data are reported in Supplementary Table S5. HC were enrolled from the Partners Parkinson’s and Movement Disorders Center and the Memory Disorders Unit at Massachusetts General Hospital. HC had no personal or family history of neurodegenerative diseases. Mean age of controls was 64.4 ± 10.7 years with a 50% male ratio. Further clinical data are reported in Supplementary Table S5. Exclusion criteria for all study subjects were age < 21 years, hematologic malignancies or coagulopathies, known severe anemia (hematocrit < 30), and known pregnancy. Regarding the sample processing, it has been stated that the procedures outlined below have been reported. The PAXgene Blood RNA Kit (QIAGEN, Germantown, MD, USA) was used for the purification of total RNA from whole blood samples collected in a PAXgene Blood RNA Tube and total RNA was extracted using the PAXgene procedure; four μg of total RNA was eventually reverse-transcribed into cDNA, biotin-labeled, purified, hybridized to the Affymetrix Human Genome U133A Array (GPL96) (Santa Clara, CA, USA), and scanned using standards Affymetrix procedures, for the final production of raw CEL files deposited on the database and used in our analysis. For further details on the cohort data, please refer to the author’s original work [17].

### 4.3. Bioinformatics Analysis

The pipeline implemented in BioTEA v1.1.0 [88] was run to download, preprocess, and perform Differential gene Expression Analysis (DEAs) with microarray data from the selected GSE6613 dataset. The first steps of the pipeline were composed by the data retrieval of raw CEL files and data preprocessing, which included background-subtraction, quantile-quantile normalization, and quality-checks on top of raw expression data. The analysis commenced with the initiation of the differential expression analysis and the establishment of specific parameters. The variables of interest, delineated by the disease state, and the targeted contrast, comparing PD samples to HC, were defined. To ensure compatibility with data generated using the Affymetrix Human Genome U133A Array (GPL96), a log_2_ expression threshold of 4 was applied. Genes meeting the criterion of a q value < 0.05 were retained, and their regulation status was determined based on whether the log_2_ fold change was positive or negative. The options and the expression matrix were eventually read and DEA was performed with the “RankProd” package, which was used to handle DEGs detection under the two experimental conditions [89,90]. Our choice to use this non-parametric statistical technique was moved by previous results obtained using rank products that have already been shown to be more biologically meaningful than the one generated by linear models, especially in studies with noisy data and a low number of replicates. The STRING (Search Tool for the Retrieval of Interacting Genes/Proteins) database [91] was employed to construct the interaction network among DEGs in our study. STRING provides a comprehensive resource for evaluating protein–protein interactions, incorporating both experimental and predicted interaction data. For the analysis, we used as an advanced setting the highest confidence (0.900) for “Required score”, and for “FDR stringency” we used the highest (1%). We used the version 12.0 of STRING accessed on 21 December 2023 through the address https://string-db.org/. The DEGs interactions retrieved using STRING were explored through Cytoscape v. 3.10.1 [92], which allowed us to perform the analysis of the network and obtain data about different network parameters. In addition to network analysis, the list of DEGs was also employed for conducting GO enrichment analysis. This analysis identified gene ontologies that exhibited statistically significant alterations. Enrichment analysis was performed using R v. 4.2.0 (R Core Team) with the package “clusterProfiler” v. 4.6.2 [93] and “biomaRt” v. 2.54.1 [94]. The list of enriched ontologies provided insights into the specific areas where transcriptomic changes were concentrated and which processes were most affected by PD.

## 5. Conclusions

Our findings support the presence of a global reduction in protein synthesis in PD and proteostasis imbalances. The precise mechanisms that trigger the deregulation of protein synthesis have yet to be fully elucidated. We hypothesize that the repression of protein synthesis may be involved in neurodegeneration through a reduction in synaptic proteins. These effects are in line with what is commonly observed in PD, particularly in dopaminergic neurons, which are particularly vulnerable to disruptions in protein synthesis and cellular stress. Our results highlight protein translation defects in PD patients that are also found in blood. The precise regulation of these events may provide insights into the molecular basis of PD. The dysregulation of proteostasis can be considered a pioneering step in the pathogenesis of PD, and the screening of proteostasis-related biomarkers can be explored for early diagnosis.

### Limits and Future Prospects

Our study provides insights into the possible role of translational processes and their reduction in PD patients. It is important to highlight that our analysis was carried out on peripheral blood data. Therefore, future studies correlating the findings with brain tissue would be necessary. Furthermore, extending our analysis to a larger sample would help improve the robustness and generalizability of our findings. By increasing the patient cohort and clinical information, future studies will allow us to better understand the molecular mechanisms underlying the disease.

Our study has provided preliminary data that pave the way for further studies that include other omics data (such as proteomics and metabolomics), and which will allow us to improve the early diagnosis and management framework of PD.

## Figures and Tables

**Table 1:** DEGs involved in enriched GO also present in our subnetwork.

Ontology	Description	Total DEGs	Subnetwork DEGs	DEGs Symbol
BP	Cytoplasmic translation	26/375	21/36	*EIF3A*/*EIF4A2*/*EIF5*/*RPL15*/ *RPL18A*/*RPL29*/*RPL3*/ *RPL4*/*RPL5*/*RPL7*/*RPL9*/ *RPS15A*/*RPS16*/*RPS17*/ *RPS21*/*RPS24*/*RPS26*/*RPS3A*/ *RPS4X*/*RPS5*/*RPS7*
CC	Ribosomal subunit	21/375	20/36	*NPM1*/*RPL15*/*RPL18A*/*RPL29*/ *RPL3*/*RPL4*/*RPL5*/ *RPL7*/*RPL9*/*RPS15A*/*RPS16*/ *RPS17*/*RPS21*/*RPS24*/ *RPS26*/*RPS3A*/*RPS4X*/*RPS4Y1*/ *RPS5*/*RPS7*
CC	Ribosome	22/375	20/36	*NPM1*/*RPL15*/*RPL18A*/*RPL29*/ *RPL3*/*RPL4*/*RPL5*/ *RPL7*/*RPL9*/*RPS15A*/*RPS16*/ *RPS17*/*RPS21*/*RPS24*/ *RPS26*/*RPS3A*/*RPS4X*/ *RPS4Y1*/*RPS5*/*RPS7*
CC	Cytosolic ribosome	20/375	19/36	*RPL15*/*RPL18A*/*RPL29*/ *RPL3*/*RPL4*/*RPL5*/*RPL7*/ *RPL9*/*RPS15A*/*RPS16*/ *RPS17*/*RPS21*/*RPS24*/ *RPS26*/*RPS3A*/*RPS4X*/ *RPS4Y1*/*RPS5*/*RPS7*
MF	Structural constituent of ribosome	20/375	19/36	*RPL15*/*RPL18A*/*RPL29*/ *RPL3*/*RPL4*/*RPL5*/*RPL7*/ *RPL9*/*RPS15A*/*RPS16*/*RPS17*/ *RPS21*/*RPS24*/ *RPS26*/*RPS3A*/*RPS4X*/ *RPS4Y1*/*RPS5*/*RPS7*

In the first column is reported the type of the enriched GO described in the second column. In the third column are reported the numbers of DEGs included in the GO versus the total number DEGs. In the fourth column are reported the numbers of DEGs included in the GO versus the number of DEGs in the subnetwork. In the last column are reported the symbols for the DEGs related to our subnetwork included in the enriched GO.

## Data Availability

The data presented in this study are openly available in the Gene Expression Omnibus (GEO) database (https://www.ncbi.nlm.nih.gov/gds; accessed on 27 November 2023), under the accession number GSE6613.

## Supplementary Materials

The supporting information can be downloaded at: https://www.mdpi.com/article/10.3390/ijms25021299/s1.
